# Design Strategies and Biomimetic Approaches for Calcium Phosphate Scaffolds in Bone Tissue Regeneration

**DOI:** 10.3390/biomimetics7030112

**Published:** 2022-08-13

**Authors:** Federico Pupilli, Andrea Ruffini, Massimiliano Dapporto, Marta Tavoni, Anna Tampieri, Simone Sprio

**Affiliations:** 1 Institute of Science and Technology for Ceramics, National Research Council of Italy, 48018 Faenza, Italy; 2 Department of Chemical Sciences, University of Padova, 35122 Padova, Italy

**Keywords:** biomimetics, hydroxyapatite, ion doping, 3D scaffolds, bone regeneration, biomineralization, biomorphic transformations

## Abstract

Bone is a complex biologic tissue, which is extremely relevant for various physiological functions, in addition to movement, organ protection, and weight bearing. The repair of critical size bone defects is a still unmet clinical need, and over the past decades, material scientists have been expending efforts to find effective technological solutions, based on the use of scaffolds. In this context, biomimetics which is intended as the ability of a scaffold to reproduce compositional and structural features of the host tissues, is increasingly considered as a guide for this purpose. However, the achievement of implants that mimic the very complex bone composition, multi-scale structure, and mechanics is still an open challenge. Indeed, despite the fact that calcium phosphates are widely recognized as elective biomaterials to fabricate regenerative bone scaffolds, their processing into 3D devices with suitable cell-instructing features is still prevented by insurmountable drawbacks. With respect to biomaterials science, new approaches maybe conceived to gain ground and promise for a substantial leap forward in this field. The present review provides an overview of physicochemical and structural features of bone tissue that are responsible for its biologic behavior. Moreover, relevant and recent technological approaches, also inspired by natural processes and structures, are described, which can be considered as a leverage for future development of next generation bioactive medical devices.

## 1. Introduction

The skeleton plays a fundamental role in human physiology for both protection and support of many vital organs. At the same time, it serves as a framework for the body, providing attachment for the muscles and fibrous connective tissues, such as ligaments and tendons. Bone tissue may be subjected to trauma or other degenerative diseases during lifetime. Indeed, the regeneration of impaired bone tissue is still a largely unmet problem, particularly when it comes to the treatment of critical size and load-bearing bone defects, which implies the occurrence of serious disabilities and impact on the human wellbeing as well as on direct and indirect healthcare costs [1]. Clinical treatment of bone defects typically involves bone grafting techniques in orthopedic, cranio-maxillofacial, and spinal surgery. For years, the use of bone autografts was considered as the most suitable choice for the purpose of bone repair and regeneration, in terms of histocompatibility and non-immunogenic properties. Indeed, the harvest of bone from the patient consists primarily of cortical and cancellous bone with the essential components to achieve bone regeneration processes, such as growth factors including bone morphogenetic proteins (BMPs) and osteoprogenitor cells. Conversely, the use of allografts is typically associated with risks of immunoreactions and transmission of infections. Due to the devitalization processes (irradiation or freeze-drying processes) following the extraction from the deceased donor, allografts have reduced osteo-inductive properties and no cellular component [2]. On the other hand, however, autografts require generally a second intervention at the site of the harvest, resulting in a very expensive and painful surgical practice associated with surgical risks, such as infection and chronic pain [3,4]. For these reasons, decades of intensive effort are dedicated to the development of synthetic bone grafts with effective regenerative ability while mitigating the risks associated with clinical treatment [5].

Despite recent technological advances in material science and an increasingly comprehensive understanding of the bone regeneration process, intrinsic limitations in current implants/scaffolds can still make the process ineffective in most cases, mostly due to the low bioactivity of these devices. Indeed, scientists are increasingly aware that the complex composition and 3D multi-scale structure of bone tissue play a synergistic and key role in cell signaling, regulating bone development and metabolism as well as its mechanical performance, which is relevant in cases of load-bearing bone defects [6]. These properties represent a target for modeling and reproducing synthetic bone scaffolds to create an appropriate, cell-instructive micro-environment, which promotes and sustains various complex and interacting biologic phenomena at the base of bone metabolism, such as:

- Stem cell adhesion on the scaffold, proliferation, and differentiation: These phenomena require hydrophilic scaffold surface and bioactive chemical composition, permitting the exchange of osteogenic signals that promote osteoblastic differentiation [7].
- The complete colonization of the scaffold by bone-forming cells, assisted by substantial vascularization: This requires scaffolds with osteoconductive ability, which is, once again promoted by the chemical composition as well as the existence of a wide open and interconnected porosity.
- The activation of osteoclastic bio-resorption, which is, once again, substantially related to the chemical composition, thus permitting the replacement of the scaffold with the newly formed bone by the natural physiologic process. In this context, nanostructured scaffold materials can offer high specific surface areas and a more chemically active surface, facilitating the bio-resorption process.
- Bone mechano-transduction, consisting of a complex cascade of phenomena that translate mechanical forces into bioelectric signals, is a major source of cell signaling, in which bone tissue is able to continuously renew and remodel over time, and to self-repair following damage of limited entity [8].

To obtain scaffolds that are able to drive all of the above phenomena as a whole, material scientists are attempting to develop materials and devices that exhibit high biomimetic character, i.e., capable of exhibiting bioactivity instructing cells by virtue of their chemical and structural similarity with the target tissue. This approach, which can be referred to as the “biomimetic concept”, requires substantial knowledge of the basic phenomena leading to new bone formation, maturation, and remodeling. However, it most relevantly requires novel scientific and technological approaches for biomaterial development to provide the new devices with physicochemical and structural features which are relevant for instructing bone-forming cells. This goal, which is increasingly pursued in recent years, is still a great challenge, due to the difficulty in mimicking the complex nature of bone tissue [9,10]. Therefore, this review paper will highlight some physicochemical and structural features of bone tissue that are responsible for its biologic behavior and will describe relevant and recent methods, including nature-inspired approaches that attempt to develop bone scaffolds with enhanced regenerative ability.

## 2. Bone Tissue: Composition and Structure

### 2.1. Bone Composition

**Figure 1:**
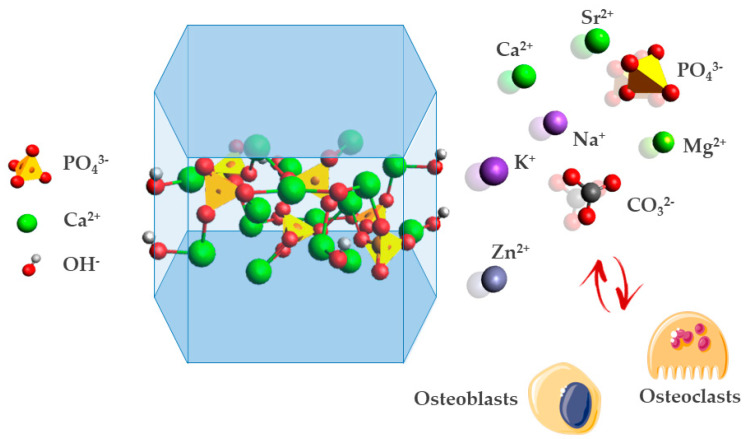
Pictorial illustration of the hydroxyapatite crystal structure and chemical signaling to bone cells.

Bone is a very complex tissue, which is mainly composed of carbonated, low-crystalline, calcium deficient hydroxyapatite, and accounts for around 65% of the total bone mass. Moreover, it is embedded in an organic matrix prevalently composed of collagen and an organic molecule disposed of as a triple helix of protein chains that present high tensile strength [11,12]. The term “apatite” generally refers to a family of compounds with similar structures (hexagonal system, space group, P63/m). The hydroxyapatite phase contains ten calcium (Ca), six phosphate (PO_4_), and two hydroxyl (OH) groups, with a Ca/P molar ratio equal to 1.67. On the other hand, biologic apatite is generally a nonstoichiometric and Ca defective phase, with Ca/P ratios varying from 1.5 to 1.67, affecting the biological properties of apatites [9]. The crystal pattern can be additionally addressed by the formula Ca1_4_Ca2_6_(PO_4_)_6_(OH)_2_, in which Ca1 defines calcium atoms disposed of a columnar arrangement traverse to the lattice and Ca2 in a hexagonal fashion (Figure 1). The six PO_4_ units are disposed of in the crystal lattice between a pair of Ca^2+^ ions in the outer hexagon when observed below the c-axis. In this peculiar structure, the hydroxyl ions are aligned along the c-axis, delimited by the Ca2 ions positioned in a series of triangles lying parallel to the basal plane. Although their confinement inside the overlapping triangles are formed by the Ca2 ions, the OH groups have relative freedom of movement along the channel formed (which has a diameter of ~3 Å), leading to specific properties related to ion mobility and proton conduction [13].

Apatites can undergo noticeable crystal lattice distortion, enabling the accommodation of foreign cations and anions that differ in size from Ca^2+^ and PO_4_^3−^, respectively. This feature has a biologic relevance since apatites in biological tissue play the role of an ion reservoir of bioactive ions or a trigger for biological signals in response to external stimuli, appointing apatites as a fascinating example of metabolic crystal. Specifically, biological apatites incorporate in their structure various bioactive ions, such as Mg^2+^ partially replacing Ca^2+^ or CO_3_^2−^ partially replacing PO_4_^3−^ (the so-called B carbonation), entailing changes in crystallinity, thermal stability, morphology, solubility, and other physicochemical and biological properties [14]. Among all these bioactive ions, the most prominent molecule present during apatite formation and evolution is water. The peculiar crystal structure of low-crystalline synthetic and biological apatites has been studied with spectroscopic techniques (Fourier transform infrared [FTIR] and ^31^P nuclear magnetic resonance [NMR]), revealing the existence of large quantities of water on the surface and sub-surface layer of apatite crystals (called hydrated layer), which are crucial for the binding of soluble non-collagenic proteins [15]. In addition, the presence of water molecules can fill ion vacancies in the crystal lattice (calcium sites or oxygen vacancies remaining in CO_3_-for-PO_4_ substitution or vacancies in the OH sites), although it is still debated and unexplored [16,17]. As water molecules are omnipresent in the apatitic crystal in humid conditions, their presence should be taken into consideration and characterized (e.g., by thermogravimetric and spectroscopic analyses) as its diminution causes substantial changes in the crystal lattice and denaturation of its protein-binding ability. Depletion of water molecules upon dehydration of bone tissue, for example, induces the decrease in spacing between collagen molecules, consequently reducing bone toughness [18]. In addition to its complex molecular structure, the biologic activity of bone is also supported by its unique 3D architecture which, owing to a hierarchical organization developed from the molecular to the macroscopic scale, provides an outstanding mechanical ability, in turn, enabling self-repair and self-adapting mechanisms [9].

### 2.2. Hierarchical Bone Organization

Bones are highly organized materials whose hierarchic architecture, with details ranging from nanometers to centimeters in size, depends on their mechanical role within the whole skeleton. Self-assembled mineralized fibrils hierarchically arranged in the presence of non-collagenous proteins and water are ultimately responsible for the mechanical properties of bone tissue, delivering at the same time nutrients and especially environmental signals to the implanted osteocytes implanted on it [19,20]. The unique structure of bone tissue is vital to activate mechano-transduction phenomena at the cell level, which is considered a major mechanism providing the bone with the ability to adapt to the loads under which it is placed, as well as to self-repair and self-regenerate upon damage of limited entity [21,22]. To understand the mechanical properties of bone tissue, it is important to underline how bone tissue behaves at various levels of its hierarchical structure [23,24]. These levels and structures can be classified in:

1. The macrostructure: Cancellous and cortical bones.
2. The microstructure (from 10 to 500 microns): Haversian systems, osteons.
3. The sub-microstructure (1–10 microns): Lamellar and woven bones.
4. The nanostructure (from a few hundred nanometers to 1 micron): Fibrillar collagen and embedded mineral phase.
5. The molecular structure (below a few hundred nanometers): Molecular structure of constituent elements, such as mineral, collagen, and non-collagenous organic proteins [25].

#### 2.2.1. The Macrostructure

An important distinction must be made between the two classes of bone tissue present in vertebrates, with specific functionalities:

- Cortical bone (also called compact bone) is a dense structure organized as regular layers of lamellae tissue, with different thicknesses depending on the location of the bone. The location of cortical tissue is on the outer layer of bone tissue, constituting about 80% of the total mass of the skeleton. Transverse sections of lamellae arrangement in load-bearing bones, such as the femur and other lower extremity bones exhibit a denser and thicker structure compared with other tissues, such as lateral and posterior cortex.
- Cancellous bone (also called trabecular or spongy bone) is less dense tissue in which collagen fibers, and therefore, lamellae are arranged in an irregular way, interconnecting and forming the trabecular tissue network. Cancellous bone is located within metaphysis and epiphysis at the end of long bones, and in short bones, as well. Metabolic activity, such as bone cell production and mineral exchange is higher in cancellous bone compared with the cortical bone as its porous structure is highly vascularized and contains red bone marrow [12,26].

#### 2.2.2. The Microstructure

Planar arrangements of mineralized collagen called lamellae (an orderly arrangement of mineralized collagen bundles) with a thickness ranging from 3 to 7 μm can wrap in concentric layers (3–8 lamellae) around canals, such as blood vessels to form a cylindrical structure known as an osteon, 100–120 μm in diameter, extending parallel to the long axis of the bone. Osteons can directly form de novo from vascularized channels, called primary osteons or can be reproduced from bone remodeling processes, called secondary osteons. Secondary osteons, also commonly known as Haversian systems, are the most abundant in mature skeletons and are characterized by the presence of a cement line as the outer layer of the osteon itself, a sign of the ceased resorption process [27].

The central canals may branch or combine with the prevailing direction, which is along the bone axis and the cement outer layer, facilitating the attachment of each series of lamellae within the lamellae bundle [19,28].

#### 2.2.3. The Sub-Microstructure

Lamellar bone is the most common material that forms bone tissue, and it is composed of a series of lamellae. From TEM and SEM micrographs, it has been established that the orientation of a series of lamellae does not follow a strict parallel arrangement of mineralized collagen fibers, but is made of highly interlaced bundles of fibers, with a different density in adjacent layers [29]. The alternation of dense and loose density layers could be a sign of the recruitment of osteocyte-differentiating osteoblast. The secretion of densely interlaced fibrillar matrix by osteoblast overlaps with the loose fibrillar matrix already laid down by pre-osteocytes during their transformation from osteoblast to osteocytes. A similar arrangement of lamellae has been found in woven bone, a transient tissue with disordered fibrous structure, built during bone remodeling processes and fracture repair. Contrarily to lamellar bone, woven bone is composed of mineralized collagen fibril bundles with little or no 3D orientation [19].

#### 2.2.4. The Nanostructure

Collagen fibers covered by heterogeneously nucleated minerals are the most prominent structures observed at a nanometric scale. The mineralized collagen fibrils consist of an assembly of 300 nm long and 1.5 nm thick collagen molecules, which are deposited by the osteoblasts (bone-forming cells) into the extracellular space, and then self-assemble into fibrils with a diameter around 100 nm. Adjacent molecules with the fibrils are staggered along the axial direction by D ≈ 67 nm, generating a characteristic pattern of gap zones with 35 nm in length and overlap zones with 32 nm in length within the fibril [30]. Immuno-histological studies have shown a preferential and periodical assembly of the constituting macromolecules into fibers [31]. Mineralized collagen fibrils in vivo self-assemble in a complex fashion, apparently starting in the osteoblast endoplasmic reticulum, then in compartments outside the cytoplasm, and finally moving to the extracellular space. The diameters of these arrays can vary from less than a micron to several microns and fibril arrays are only present in the ordered material. Most fibrils in the disordered material appear as individual fibrils and show little preferred orientation compared with the ordered material [19].

#### 2.2.5. The Molecular Structure

Mineralized collagen fibrils at the nanometric level are composed of the three main materials: Crystals, collagens, and non-collagenous organic proteins. Plate-shaped crystals occupy periodical spaces within the collagen fibrils, limiting their crystal growth and therefore their dimension at a nanometric scale, with average lengths and widths of the plates at about 50 × 25 nm and crystal thickness of 2–3 nm [25]. The mineral crystals, composed of mainly apatite with small impurities, such as Na, K, Mg, HPO_4_, and carbonates, grow with c-axes of the crystal roughly parallel to the long axes of the collagen fibrils [32]. While the X-ray diffraction pattern is the HA, the near absence or absence of the hydroxyl group has been proven repeatedly by chemical methods and FTIR, 1D-NMR, and 2D-NMR spectroscopy [17,33]. The primary organic component of the matrix is Type I collagen, secreted by osteoblasts and self-assembled into fibrils with a specific tertiary structure. Non-collagenous organic proteins, including phosphoproteins, such as osteopontin, sialoprotein, osteonectin, and osteocalcin, may function to regulate the size, orientation, and crystal habit of the mineral deposits. Through chelation of calcium or enzymatic release of phosphorus from these proteins, they may serve as a reservoir for calcium or phosphate ions for mineral formation [34].

## 3. Bone Tissue: Formation and Metabolism

### 3.1. Mineralization Process of Bone Tissue

Bone formation in mammals follows a complex cascade of phenomena known as biomineralization. This physiological process is regulated by interactions of apatite precursor minerals, such as octacalcium phosphate Ca_8_H_2_(PO_4_)_6_·5H_2_O (OCP) and amorphous calcium phosphate Ca_3_(PO_4_)_2_·nH_2_O, *n* = 3–4.5 (ACP), in addition to organic extracellular molecules that drive the formation of hierarchically structured hybrid organic–inorganic tissues. Several control mechanisms activated by the bio-organic matrix regulate the formation and organization of the mineral phase at the multi-scale. This guides the formation of nanoplate-sized HA parallelly-oriented to the collagen fibril axis structures with a complex hierarchical organization, that is able to withstand mechanical strain and adapt to constant external stimuli [30,35]:

- Chemical factors: Precipitation of ions naturally present in the environment, mediated by complex macromolecular organic structures, which act as sites of heterogeneous nucleation and control specific chemical interactions.
- Spatial factors: Confinement of the nuclei growth, as well as a constraint in their shape and contact with the organic substrate.
- Structural factors: Inducing peculiar crystallographic features driven by the interaction between the mineral phase and the organic template.
- Morphologic factors (morphogenesis): Where the mineral phase takes place in a complex architecture on a macroscopic scale, strictly dependent on the combination of the various phenomena above-mentioned, which hierarchically occur on different dimensional scales in correspondence with the sites of heterogeneous nucleation [36].

The formation of calcium phosphate (CaP) phases in bone tissue includes the sequestration of calcium and phosphorous ions into ACP stable precursors, and the templating of these clusters into ordered forms [37,38]. Formation and evolution of relatively stable amorphous phases have been studied to perform an in-depth examination of biomineralization processes. In vitro studies monitored with micro-Raman spectroscopy combined with solid state NMR, for example, have been used to show that ACP is first formed and subsequently transforms into OCP, then progressively turns into carbonated apatite [39,40]. Mineralization evolution of apatite precursors is further regulated by the presence of non-collagenic proteins, such as albumin and osteocalcin. OCP crystallization studies in vitro in the presence of albumin prove that significant localized variations in albumin concentration in body fluids can effectively inhibit crystal growth, especially during the early stages of biomineral formation [41], while osteocalcin is found to be attached to the collagen structure and interacts with the Ca-sites, participating in the alignment of apatite crystallites during mineralization [42,43].

Development and better understanding of mineralized fibril formation have also been focused on the re-evaluation of the role of collagen during biomineralization. Collagen was long believed to only serve as a structural matrix and considered to be inactive in biomineralization. Recently, collagen was discovered to play an active role in templating apatite mineralization process by the discovery of a positively charged region existing in collagen fibrils at the interface of the gap and overlap [37]. Cryo-TEM studies of collagen fibrils mineralization showed, for example, that once ACP particles enter the fibril structure, collagen with its charged amino acid groups acts as a nucleation site for the formation of nanosized apatite [44]. Despite extensive efforts to understand biomineralization processes, to date, only simplified biomimetic structures have been achieved.

### 3.2. Bone Modeling and Remodeling Processes

A peculiarity of bone tissue relies on the fact that its overall structure changes in response to physiological or mechanical forces undergoing modeling and remodeling processes. The dynamic behavior of bone tissue is crucial for its distinctive mechanical and biological properties, and is controlled by the independent action of osteoblasts and osteoclasts cells, two main cell lines responsible for bone turnover. Therefore, a key aspect in the design and development of regenerative biomaterials is the ability to promote and sustain this biologic phenomena. As a result, this enables the instruction of osteoblasts and osteoclasts cells, which is a fundamental aspect for the metabolism and maturation of the newly formed bone tissue [45,46]. More specifically, on the one hand, the composition and structure of bone scaffolds should favor stem cell adhesion and differentiation into osteoblasts. On the other hand, the bone scaffold should allow osteoclastic resorption which, in contrast with chemical dissolution, permits the balance of the kinetics of scaffold bio-resorption with new bone formation. In this context, in vitro studies can easily assess whether the scaffold has the potential to promote this complex biochemical pathway. However, more significant results can be obtained with bioreactor studies, as the cell response in a 3D dynamic environment is considerably more predictive of the actual cell behavior and fate in vivo [47].

- Osteoclasts are highly specialized cells that are known to be capable of resorbing bone tissue, derived from mononuclear monocyte-macrophage precursor cells. In addition, RANKL and macrophage CSF (M-CSF) are two cytokines produced by marrow stromal cells that lead to osteoclast formation. Osteoclasts bind to bone matrix via integrins (transmembrane receptors that facilitate cell–cell and cell–extracellular matrix adhesion [48]) in the osteoclast membrane linked to bone matrix peptides. The β1 family of integrin receptors in osteoclasts binds to collagen, fibronectin, and laminin, but the main integrin receptor facilitating bone resorption is the α_v_β_3_ integrin, which binds to osteopontin and bone sialoprotein [49]. Bone resorption depends on osteoclast secretion of hydrogen ions and cathepsin K enzyme, which define the active resorption process in two distinct phases: Acidification and proteolysis. Hydrogen ions, secreted by the osteoclastic vacuolar adenosine triphosphatase (V-ATPase) channels, acidify the resorption compartment called the “sealing zone” and dissolve the mineral component of the bone matrix, coupled with the transport of chloride ions via an electrochemical gradient [50]. The proteolysis occurs when cathepsin K digests the organic matrix, which is mostly composed of type I collagen [51,52].
- Osteoblasts are the cells responsible for the formation of bone tissue, derived by the differentiation of bMSC recruited from bone marrow to the bone surface by cytokines and growth factors, for example, transforming growth factor-β and insulin-like growth factor 1, which are released from the bone matrix during the resorption phase [53,54]. Osteoblasts express high levels of alkaline phosphatase (ALP) and osteocalcin and secrete large quantities of type I collagen and other specialized matrix proteins, which form osteoid tissue. The organic matrix acts as a template for the deposition of the mineral inorganic phase of HA. Interaction of osteoblasts among themselves, with lining cells, and with bone marrow cells are established by adherent junctions, tight junctions, and gap junctions. Adherent junctions, mainly mediated by cadherins, in addition to tight junctions serve to join cells and facilitate their anchorage to the extracellular matrix (ECM) [55].

Therefore, bone modeling is defined as the process of bone formation by osteoblasts (called formation modeling) or bone resorption (called resorptive modeling) by osteoclasts on a given surface. Bone modeling processing occurs solely on pre-existing bone surfaces and has the function of increasing bone mass and altering bone shape. During bone modeling, osteoclast and osteoblast activity are not strictly coupled, thus the two processes occur in a relatively independent manner of one another [56].

Bone remodeling, on the other hand, is the process by which bone is renewed to maintain bone strength and mineral homeostasis and it is characterized by the sequential action of both osteoclasts in bone resorption and osteoblast in bone formation in the same spatial location. Remodeling involves continuous removal of discrete packets of old bone, replacement of these packets with a newly synthesized collagen matrix, and subsequent mineralization repairing micro-damages present on the surface of bone tissue. The remodeling cycle is composed of four sequential phases [12,57]:

- Activation: Osteoclast precursors lay on the bone tissue surface and differentiate to mature and functional osteoclast.
- Resorption: Osteoclast cells stick on the surface of the mineralized matrix to begin the process of bone resorption. The results of bone tissue dissolution are released in body fluids, such as blood and urine, to be useful biomarkers for the next resorption steps.
- Reversal: Osteoclasts interrupt bone tissue resorption and osteoblasts begin bone formation. Although the mechanism is still under investigation, direct cell–cell interaction between osteoclasts and osteoblasts (or their precursors) may trigger the disruption of one process and the initiation of the other [58].
- Formation: Osteoblasts lay down an unmineralized organic matrix (osteoid), which is primarily composed of type I collagen fibers and serves as a template for inorganic HA crystals [59].

After the remodeling cycle, most of the osteoblasts involved in bone remodeling processes die through apoptosis, while a small percentage is incorporated into the osteoid tissue and actively become osteocytes or remain at the bone surface as inactive bone cells, as they could be activated at any moment in subsequent bone remodeling processes [54].

### 3.3. Osteoinductivity and Osteoconductivity

As previously stated, one of the main properties to be exhibited by bone scaffolds is the ability to recruit immature stem cells and lead their differentiation process into osteoblasts. This process is called osteoinduction. Various osteogenic agents including transforming growth factor-beta (TGF-β), BMPs, and other growth factors via related signaling pathways are fundamental molecules throughout this osteoinduction process [60]. CaPs, such as hydroxyapatite (HA), tricalcium phosphate (TCP), and biphasic calcium phosphate (BCP) are clear examples of osteoinductive biomaterials as they show pronounced adsorption and differentiation ability, enabling better bone regeneration without the addition of cells or growth factors [61]. In this context, synthetic CaP-based materials can offer bio-available inorganic ions, particularly when developed as nanocrystalline phases, providing at the same time a suitable environment for proliferation and differentiation of bone-resorbing cells, making it the best candidate in the design of biomimetic scaffolds [45].

A comparison of the behavior of bioceramic CaPs observed in various animal models shows a trend in improved osteoinductivity behavior in mouse > dog > rabbit > rat [62]. Although mice could be the best choice to represent osteoinductive processes as they have several advantages in terms of economic impact, convenience, and osteogenesis ability, it should be noted that the osteogenesis mechanism is substantially variable and related to the chosen animal model, as well as to the source of stem cells, the type of signaling molecules, and transforming factors [63,64,65].

As fairly connected with osteoinductivity in terms of bioactivity, osteoconductivity is the ability of bone tissue to grow along an implanted material surface [61]. Various factors have effects on the osteoconduction process, which depends on the presence of defect sites as well as morphological factors. In general, SEM micrographs are used to characterize bone coverage of bioactive materials, with numerical models able to simulate osteoconduction processes [66,67]. As fairly connected with osteoinductivity processes, the osteoconduction of implant surfaces depends on the action of differentiated bone cells, that may originate in pre-existing, pre-osteoblasts/osteoblasts that are activated by trauma or in differentiated mesenchymal cells activated by osteoinduction processes [68]. Bone formation along an implanted material involves the action of various types of bone growth factors, for example, insulin-like growth factor (IGF I, II, also called somatomedins) fibroblast growth factor (FGF), TGF-β, and platelet-derived growth factor (PDGF) [69,70], as well as a proper vascularization along its surface [71]. Osteoconductivity behavior is experimentally observed by SEM micrographs of bone tissue coverage on the implanted material, with more tissue and bone cells grown on the bone graft, indicating a more pronounced osteoconductivity [72]. Osteoconduction processes could potentially occur on the surface of a wide variety of materials that often are not regarded as ideal from the point of view of biocompatibility. In this context, osteoconduction processes may occur with or without resorption of the implanted material. In addition, as bioactive materials exert their regenerative function within a three-dimensional (3D) environment where internal bone tissue ingrowth and vascularization occur, it is necessary to assess effective osteoconductivity as depending not only on the chemical and compositional affinity with biological apatites, but also with specific morphological properties (for example, porosity extent as well as its 3D organization and interconnection) that permit natural bio-resorption processes [5].

## 4. Translation of the Biomimetic Concept to 3D Scaffold Development

### 4.1. Limitations of Current Approaches and Further Challenges in Tissue Engineering

As already stated, one of the main targets in the design of biomimetic CaP ceramic scaffolds is the ability to create a micro-environment that stimulates cell differentiation into osteoblasts and to stimulate cell chemotaxis and new bone matrix deposition. Although HA has many advantages in terms of bioactivity and bioavailability, it is limited by fragility, typical of ceramic materials, poor mechanical strength, and thus, inability to withstand severe load-bearing conditions. One of the most common ways to confer mechanical properties to CaPs (and ceramic phases in general) is via densification processes obtained through high temperature sintering processes. Although densification usually yields ceramic bodies with superior mechanical strength, the properties of CaP materials in terms of crystallinity, grain size, porosity, and composition vary significantly upon sintering. High crystallinity, low porosity, and small grain size tend to provide higher stiffness, compressive strength, and toughness, but hamper tissue growth and binding of bone-resorbing cells [73].

Sintering temperature of synthetic HA in a range between 950–1150 °C causes a general increase in grain size, density, crystallite size without a significant change in the crystallographic, and the Ca/P atomic ratio. Upon heating to 1250 °C, samples undergo several structural transformations with the formation of α- and β-tricalcium phosphate (TCP: Ca_3_(PO_4_)_2_) that, although biocompatible, are not found in bone [74]. The situation further complicates when non-stoichiometric HA, but rather calcium-deficient or biomimetic ion-doped HA, are considered. Studies conducted on Mg- and Sr-substituted HA prove that the introduction of Mg ions, at a weight percentage of 0.5% provokes the formation of both β-TCP and CaO upon thermal treatments, in contrast with what occurred in pure HA, which did not dissociate in secondary phases. These phenomena could be asserted to the distortion effect of the crystal lattice caused by the substitution of calcium with foreign atoms, which ultimately may lead to dissociation in secondary phases upon thermal treatment, with the amount of secondary phases increasing by raising the working temperature [75,76].

Problems related to the formation of secondary phases during the densification processes, in association with reduced biological activity, substantially limit the regenerative effectiveness of sintered scaffolds in bone tissue engineering. In the last decades, studies have been conducted to explore sintering behavior under microwave heat treatments rather than conventional sintering. Microwave-based approaches allow for the attainment of consolidated scaffolds with controlled structure, high densification extent, and fine grains, with a significant increase in mechanical strength with respect to conventional sintering, in addition to a higher solubility with respect to conventional sintering, leading to significant cell adhesion, distribution, and proliferation in vitro [77].

Another common strategy that has been largely used in bone scaffold development is the achievement of porous scaffolds with a 3D arrangement blending CaPs in addition to biodegradable polymers. In this context, 3D printing has become a widely used technique to fabricate composite scaffolds in regenerative medicine and is defined as a computer-aided transfer process to pattern and assemble materials with a prescribed 2D or 3D organization to fabricate bio-engineered structures with precisely designed macro-architectures [78]. Apatite powders on their own do not possess consolidation behavior that enables printability, thus it is necessary to optimize bio-inks to achieve final devices with appropriate mechanical integrity through the combination of CaP powders with polymer materials, as they possess sufficient mechanical properties and are suitable for the repair of critical bone defects [79]. In this latter aspect, various biocompatible polymers, such as polylactic acid (PLA), poly(lactic-coglycolic acid) (PLGA), and polycaprolactone (PCL) have been used for the fabrication of bone implants and even received approval from the US FDA as materials for 3D printing of biomedical implants [80]. Among them, polylactic acid (PLA) has been defined as a biomaterial with potential clinical applications in many studies due to its slow degradation properties and reliable biocompatibility [81]. Studies have been focused on the fabrication of composite nano-HA/PLA and characterization of the mechanical properties, in vitro biocompatibility, and in vivo experiments in a rabbit femoral defect model for 3 months, showing good biocompatibility and osteogenic induction ability by simulating organic and inorganic components of bone tissue, and with the potential to repair critical bone defects [82]. Nevertheless, although promising in terms of biocompatibility, one main concern of the use of these polymers in bone tissue engineering is that their intermediate degradation products (specifically, lactic acid and/or glycolic acid) by non-enzymatic hydrolysis of ester bonds in their backbone reduces the local pH, which in turn can induce inflammatory reactions and bone cell damaging at the implant site [10]. Moreover, the rapid drop of pH in vivo may accelerate the polymer’s degradation rate, thus resulting in the dispersion of inorganic particles in vivo and also in the premature loss of mechanical properties before new bone formation occurs. Another drawback related to the use of polymers added with inorganic particles is the possible loss of plastic behavior under loading, in order to be ineffective in terms of reinforcement and actually result in a decrease in fracture strength [82,83,84].

**Figure 2:**
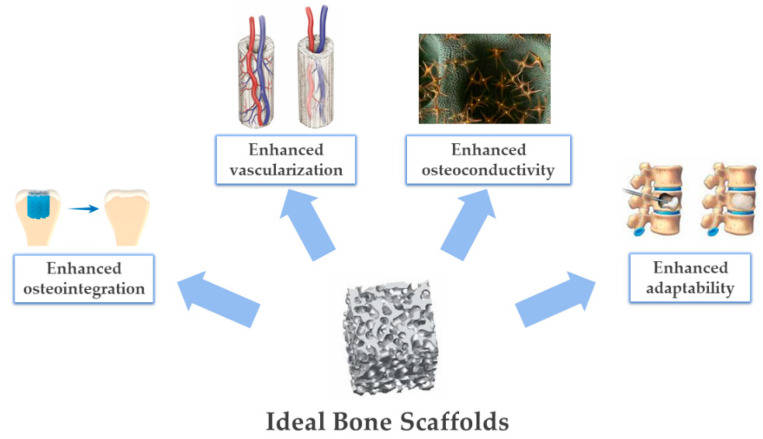
Relevant challenges in the development of biomimetic scaffolds for bone regeneration.

The above-mentioned discussions are main examples of drawbacks that can make bone regeneration ineffective, particularly while targeting large bone defects [10]. Indeed, the above-described approaches make use of materials that are not quite close to those found in natural bone, both since sintered apatites do not show the typical composition and crystallinity (and consequently, not even the required bioactivity) of biological apatites and since the polymers used have low chemical affinity with the natural bio-polymers forming biological tissues. This leads to the importance of pursuing and achieving novel effective technological solutions targeting enhanced biomimicry as a source of regenerative ability. This latter is intended as the capacity of a scaffold to exert an instructive guidance for cells in terms of both chemical and structural signals which, as highlighted in the following paragraphs and in Figure 2, aim to result in the simultaneous occurrence of various biologic phenomena.

### 4.2. Guiding Bone Regeneration by Chemistry and Crystal Structure

Hydroxyapatite is the most stable and the least soluble CaP and it is considered as the main inorganic model for the design of suitable biomedical materials due to its similarities to bone and tooth enamel. As previously stated, the crystal lattice of apatites is characterized by a loose-packed structure that permits the accommodation of foreign atoms in replacement of Ca and P. This generates surface irregularities and structural defects that ultimately result in an increased biosolubility due to the fact that the hydrolysis rate increases at lower Ca/P ratios [45,46,85]. The accumulation of defects generates lattice distortions and describes why the crystallinity of ion-doped HA is usually, yet not always, poorer than its pure analogue. The lattice disorder induced by ionic substitutions is the key factor at the basis of enhanced functionalities of biomimetic apatite phases, enabling the increase in the surface charge and consequent ability of prolonged exchange of bioactive ions, such as Ca^2+^, Mg^2+^, and HPO_4_^2−^ with the surrounding biological environment, which triggers bio-specific chemical signals active in bone regeneration [86].

For the above reasons, a main strategy to activate bioactive chemical signaling that mimics the ability of bone mineral is the achievement of low-crystalline, bioactive apatites by the introduction of doping ions into its crystal lattice. In the design of biomimetic scaffolds, Ca^2+^ sites may be occupied by divalent or monovalent cations, such as Na^+^, K^+^, Mg^2+^, Sr^2+^, Zn^2+^, Ba^2+^, Cu^2+^, and Fe^2+^, whereas PO_4_^3−^ could be substituted by atoms, such as CO_3_^2−^ and SiO_4_^4−^, while hydroxyl (OH^−^) may be replaced by CO_3_^2−^, F^−^, Cl^−^ or even be left vacant [87]. Table 1 lists the main effects of the most important doping ions in the design of biomimetic apatites:

Among all substitutions, the most prominent and studied in both biological and biomimetic tissues are the effects of carbonation. Biological tissues, such as bone, dentin, and enamel express a substantial difference in carbonate content, influencing their solubility in a biological environment. Mineral structures present in dental tissue, for example, introduce a crystal strain and increase solubility, thus dentine mineral is considerably more soluble than enamel mineral and both are considerably more soluble than stoichiometric HA [14]. The considerable differences in structure between dentine and enamel influence the interactions of the tissues with acid solutions, thus the relative rates of dissolution do not necessarily reflect the respective solubilities [102].

CO_3_^2−^ ion substitution into the apatitic crystal lattice has been shown to considerably enhance the dissolution rate and improve the solubility behavior compared with stoichiometric HA [14,101,103]. In vitro studies have found an increased collagen production by human osteoblast cells on carbonated HA compared with undoped HA, as the secretion of type I collagen strongly depends on extracellular calcium concentrations that resulted in a remarkably higher cell culture medium containing carbonated HA [104].

Magnesium plays a key role in bone mineralization and bone as it improves the osteointegration and osteoblasts activity and accelerates bone ingrowth, enhancing integrin ligand binding and protecting cells by oxidative stress [105,106]. The presence of Mg^2+^ ions on the apatite surface results in an increase in the amount of water molecules adsorbed on the surface, affecting cell attachment and differentiation [107,108].

The incorporation of Sr^2+^ ions into the apatite lattice results in an increase in the osteointegration process. It was demonstrated that the incorporation of Sr^2+^ ions into the apatitic crystal structure promotes osteoblasts differentiation and proliferation, enhancing the activity of alkaline phosphatase (ALP), as well as the production of collagen type I and osteocalcin [109]. Thanks to the ability to reduce the proliferation of osteoclasts by hindering their activity, the presence of Sr^2+^ in the apatite lattice is directly linked to the treatment of osteoporotic patients in case of tumor surgery incision or trauma [9,110]. In addition, studies have shown that the partial substitution of Ca^2+^ with Sr^2+^ can stabilize the apatitic crystal lattice, also enhancing the scaffold hardness and the mechanical stability of the newly formed bone [111,112]. Although in tissue engineering ion-doped apatites with tailored crystal structure can be very relevant in mimicking the behavior of biological apatite, ion-doped apatites in the form of nanopowders, which can be usually obtained in laboratory, are poorly useful in bone regeneration procedures, particularly when large bone defects are to be treated. In this context, a further step is required in the development of 3D scaffolds to retain cell-instructive ability and appropriate porous structure and mechanical properties.

#### 4.2.1. Synthesis Processes for the Production of Biomimetic Apatites

The use of HA in clinical application has been extensively investigated for more than four decades. HA is commercially available from a natural source or as synthetic product. Various methods were developed for the synthesis of crystalline HA phases, such as solid–state reactions involving the mixture of β-TCP and Ca(OH)_2_ powders in specific ratios in water, followed by wet milling, casting into bodies, drying, and sintering at high temperature (typically at least 1000 °C). However, the use of high temperature during these sintering treatments makes this method not ideal for the achievement of biomimetic, nanocrystalline, and bioactive apatites [74,75,76]. Therefore, various wet processes have been developed in the last decades, such as precipitation, hydrothermal, and sol-gel methods. With these methods, physical and morphological characteristics of synthetic apatites can be tailored by regulating the process conditions during the synthesis (pH, reaction time, temperature, concentration, type and state of precursor, etc.) [9,113].

- The precipitation process is the most common aqueous synthesis method used to produce HA powders and it is performed at atmospheric pressure, low temperature, and inside a reaction vessel. Precipitation typically involves a reaction between orthophosphoric acid and dilute calcium hydroxide, with the former added drop-wise under continuous stirring. Precipitation occurs at a very slow rate and the reaction temperatures can be varied between 25 and 90 °C, which is suitable for tailoring the crystallinity of the final product [114,115].
- Sol-gel materials can be manufactured by gelation of colloidal powders, hypercritical drying or by controlling the hydrolysis and condensation of precursors, then incorporating a drying step at room temperature [113,116]. Sol-gel methods are generally preferable for the achievement of apatitic nanopowders with chemical and morphological uniformity. However, the use of expensive reactants and a general difficulty in hydrolyzing phosphate sources may limit the use of this technique for large scale production [117,118].
- Hydrothermal methods involve the reaction between calcium and phosphate solutions at very high pressures and temperatures (typically in a range between 60 and 250 °C), enabling the development of well-crystallized HA particles [119,120]. Crystallinity and ion content under hydrothermal treatments can be modulated by acting on the temperature and the ionic strength, thus enabling multiple doping [121].

#### 4.2.2. Tailoring the Dissolution Mechanism and Solubility of Apatites

As previously mentioned, bone modeling–remodeling processes take place after the attachment of osteoclasts on the surface of bone tissue/implants, followed by resorption occurring through dissolution of the inorganic phase and the enzymatic degradation of the organic bone matrix. When designing devices for bone regeneration, the ability to undergo osteoclastic resorption is a key property. The bone mineral is a major actor in this context, and thus it represents a model for material scientists, as chemical–physical parameters observed in biological apatites, such as crystallinity, foreign ion content, and crystal morphology all entail more effective and different solubility behavior, which in turn affect the response of both the osteoclasts as bone-resorbing cells and osteoblasts as bone-forming cells [9]. Multiple models have been formulated to exemplify biodegradation in bone remodeling processes, simulated by the dissolution of apatites in an acidic environment [122,123]. This general mechanism takes into consideration the perfect apatitic crystal and breaks the mechanism down into several steps:

- After the placement of HA in an acidic aqueous solution, adsorption of water molecules and acid ions takes place, with the formation of a solid–liquid interface. The transport of H^+^ and A^n−^ occurs through the Nernst diffusion layer.
- The adsorption of H^+^ and A^n−^ ions on the apatite surface results in the formation of various complexes [124,125] and the protonation of orthophosphoric and hydroxyl groups. As hydroxyl groups have higher basicity and mobility in their crystalline structure than ≡POH surface groups (“≡” stands for the surface), adsorption processes usually occur faster and later diffuse away from the crystal into the bulk solution.
- The hydroxyl ion detachment from the crystal surface leaves the crystal with calcium and orthophosphate groups, that cannot be further protonated due to charge repulsion. As an electrical double layer with a positive charge on apatite cannot be continuous at the atomic (ionic) scale, the detachment of calcium atoms and their diffusion into the bulk solution is favored [123].
- A dissolution nucleus is formed after the detachment of calcium, with the formation of multiple crystal vacancies for Ca^2+^ and PO_4_^3−^ [126]. In addition, the detachment caused the formation of a charge vacancy, which is immediately compensated by the addition of protons from the acidic solution [127].
- The removal of each calcium results in decreasing attraction forces between the nearest (to calcium) orthophosphate group and the remaining part of the crystals since calcium occupies definite lattice positions, favoring the detachment of the remaining orthophosphates [128].

This simplistic model takes into consideration perfect crystalline structures with a smooth surface (dissolution steps are absent), where the detachment of one or several ions results in the formation of dissolution nuclei. Surface irregularities and structural defects all may act as a nucleus of the subsequent dissociation process [9]. As a way to mimic the resorption behavior of bone mineral, the study of biomimetic apatites with tailored properties has been widely studied through the insertion of structural defects upon ion substitution. In this context, some studies have investigated the solubility behavior of ion-doped and multi-doped apatites, considering that each ion has limited solubility in the apatite lattice, thus resulting only in partial substitution. Although the insertion of foreign bioactive ions, such as Mg^2+^, Sr^2+^, and CO_3_^2−^ ions in the apatitic structure causes a general enhancement of the overall solubility in synthetic biological fluids or in cell culture media [86,90,105,129,130,131,132,133,134], the solubility behavior of doped and multi-doped apatites can be quite difficult to predict. In fact, the extent of ion substitution in apatites is subjected to various and interlacing key aspects and the co-existence of different foreign anions and cations can influence substantially the solubility behavior of the resulting matrix. First, synthesis processes conducted at a relatively low temperature (such as body temperature) yield apatitic phases with limited crystal growth, which favors ion doping. On the other hand, ion substitution is facilitated when no charge imbalance occurs, such as, for instance, with divalent ions, such as Mg^2+^, Zn^2+^ or Sr^2+^ replacing Ca^2+^. Conversely, trivalent ions, such as Fe^3+^ or Ga^3+^ can be more easily hosted within the surface, non-apatitic layer [135,136]. Ions populating this disordered non-apatitic layer are likely less tightly bound to the apatite structure and can be more easily released and even exchanged within a fluid environment [10]. For instance, previous studies found that the simultaneous presence of Si^4+^ (replacing PO_4_^3−^) and Mg^2+^ (replacing Ca^2+^) in synthetic apatites mutually limits their release in simulated body fluid (SBF) [92]. This phenomenon suggested the formation of a complex containing these two ions, but it was not possible to precisely determine its nature nor its location within the overall apatite structure. In a general way, the few studies conducted to date on the ion release from apatites converge on some aspects, such as the relevance to have B-site carbonation and to achieve multiple ion doping. However, more systematic approaches are required to elucidate the fine physicochemical mechanisms determining the ion doping extent and particularly to the mutual interaction between different ions competing to enter in the same crystal sites. In this context, an accurate control of synthesis parameters driving kinetic and thermodynamically-driven phenomena relevant for the formation of the apatite crystal, can be a tool to better control the formation of crystallographic defects and the attainment of ion-doped apatites with more precise design of their chemical composition [137].

### 4.3. Guiding Bone Regeneration by 3D Scaffold Architecture and Porosity

Guiding bone regeneration first at the surface, and then in the bulk of synthetic scaffolds is one of the main challenges during current clinical treatments. In this context, it is necessary to take the 3D bone structure as an example and recreate its morphological features in synthetic scaffolds, controlling the surface structure and scaffold porosity. Bone macrostructure presents a substantial difference in porosity, progressing from the compact cortical bone, where porosity ranges from 5% to 10%, toward the inner cancellous bone, which is more porous, from 50% to 90% [138]. Studies conducted on HA scaffolds with parallel cylindrical pores of various sizes without interconnecting fenestration between adjacent pores showed good results in terms of osteoconduction at average pore size around ϕ50 μm, with optimum conditions at ϕ300 μm [139]. The overall porosity has a direct impact not only on cell attachment and their differentiation into bone reforming cells, but also on the rate of vascularization after implantation. Lack of pore interconnection in a 3D scaffold, for example, could inherently affect the overall bioresorbability of the scaffold itself, as bone-resorbing cells cannot penetrate and attach to the whole inner scaffold area and consequently remain only on the outer surface [140,141].

As fairly connected with osteoinduction and osteoconduction, the adherence of the implanted scaffold on the surrounding bone tissue is very relevant in tissue engineering. In this context, osteointegration refers to a phenomenon where an implant becomes intimately connected with bone in order to be unified as a whole, making this phenomenon necessary for long-term stability [142,143,144]. Porous apatitic scaffolds exhibit good mimicry of the bone mineral composition, promoting formation of new bone and a tight bone–implant interface within weeks from surgery. Highly porous structures further facilitate extensive bone penetration throughout the whole scaffold, with excellent osteointegration and positive effects on the overall biomechanical performance [145]. In vivo studies on mandibular sheep defects filled with HA scaffolds with defined macro-porosity proved that homogeneous, interconnected pores favored the formation of interpenetrating matrices of newly formed bone, thus leading to better integration and functionality of the construct [141]. The attainment of synthetic grafts with ordered, channel-like porosity from a clinical perspective may increase cell seeding efficiency and the distribution of viable cells in the inner part of the scaffolds by improving fluid conductivity and permeability, assisting in the prevention of necrotic regions formation [146]. Studies have proven that channel-like ordered porosity was found to promote the regrowth of osteon structures, whereas randomly-oriented porosity was more likely to favor the formation of new woven bone [139,147]. In addition, as the newly formed bone tissue needs nutrients and oxygen in order to subsist resorption processes, ordered interconnection is needed for internal vascularization. For this purpose, there are also other factors to take into consideration other than the overall porosity of the biomaterial, such as pore shape, interconnection, and arrangement, since they are possibly the limiting factor in bone osteoconductive processes and are determinants for optimal cell migration and blood vessel ingrowth [6].

It should be noted that, although higher porosity drastically increases bone reforming processes, biomaterials for implantations with high porous structures suffer from poor mechanical properties in terms of compressive strength and elastic modulus, which cannot meet the requirements of long-term orthopaedic applications [148,149]. For this purpose, material scientists are called to the difficult task of balancing osteoconductivity, vascularization ability, and mechanical performance by tailoring the porosity extent as well as the pore size distribution and interconnection to achieve mechanical strength, which is sufficient for early in vivo loading upon implantation and elastic properties close to those of bone. Then, osteogenic and osteoconductive ability should permit the formation of well-integrated new bone, thus progressively recovering the natural mechanical functionality of bone tissue. This is relevant to actively respond to complex biomechanical loads and activate mechano-transduction phenomena, a fundamental aspect guiding the formation and remodeling of new mechanically-functional bone [147]. All of these aspects will be considered in the next chapter, illustrating some recent approaches to develop biomimetic 3D bone scaffolds.

## 5. Recent Approaches Yielding Biomimetic Ceramic-Based Scaffolds

### 5.1. Organic/Inorganic Scaffolds by 3D Printing

Biomimetic strategies focus on the structure and components of bone tissue as models and recreate its features in synthetic scaffolds. The incorporation of natural polymers, such as collagen results in an ideal strategy in the development of bioactive organic/inorganic composite scaffolds. Three-dimensional printing techniques have been extensively investigated for this purpose. In previous studies, collagen was used in blends with biodegradable PLA and recombinant human bone morphogenetic protein-2 (BMP-2), showing comparable results with autografts for spinal fusion surgeries [150,151,152]. In another study, PCL embedding HA nanoparticles and blended with collagen showed good printability and cell proliferation proportional to the PCL content [153]. In addition, 3D printing was used to obtain biocomposites consisting of fibrillated collagen, tricalcium phosphate particles, and human umbilical cord serum (hUCS), with promising results in terms of osteogenic activity in vivo [154].

As previously mentioned, one of the main problems associated with the use of biodegradable polymers in the development of biomimetic scaffolds, such as PLA, PLGA, and PCL is related to degradation problems that could affect the overall osteointegration process. Furthermore, the highly viscous nature of polymers can lead to technological issues related to inhomogeneous infiltration, inappropriate pore interconnectivity, as well as a significant reduction in the overall porosity [155], which may hinder vascularization of the resulting composite material and also affect the final mechanical performance [156,157]. In this context, elastomer materials, such as poly(glycerol sebacate) (PGS) were recently investigated to more closely approach the mechanical properties of biological tissues [158,159,160]. Further introduction of polyethylene glycol (PEG) has been addressed to increase hydrophilicity and the resulting cell adhesion, proliferation, and differentiation on the scaffold surface. PEGylated PGS (PEGS) modified polymers have been infiltrated in CaP multiscale porous scaffolds leading to CaP/PEGS hybrid scaffolds, which still presented an hierarchically porous structure. Improved mechanical behavior and enhanced effects on in vitro cellular responses, as well as further in vivo experiments have been observed by simultaneously adjusting the polymer-coating amount and PEG incorporation in coating PGS [157]. In a further experiment, the incorporation of urethane-based PEGylated poly(glycerol sebacate) (PEGSU) in ceramic bio-inks results in freestanding hyperelastic bioscaffold, which is able to fix specific bone defects in craniomaxillofacial districts with good aesthetic results. Despite the fact that these polymeric components do not reproduce the biological features of natural polymers, such as collagen, their use can help in modulating the rheologic properties of bio-inks and facilitate micro-extrusion processes, yielding scaffolds with complex shape and geometry [161].

### 5.2. 3D Hybrid Scaffolds Using Natural Polymers and Bio-Inspired Mineralization Processes

Three-dimensional printing approaches are increasingly attempted, also pinning on the technological advances in 3D printing equipment. However, their use is still quite limited with natural polymers, particularly when containing mineral phases, due to the difficulty in obtaining appropriate rheological behavior. The use of various natural polymers in the development of composite scaffolds with enhanced bioactivity has been largely studied (for example, collagen, glycosaminoglycans, cellulose, and gelatin) [46].

Cellulose-based scaffolds were studied for their ability to nucleate bioactive calcium phosphate crystals in vitro, showing that cellulose scaffolds could be used as a model for in vitro studies [162]. In a different study, the electrospinning of gelatin blended with poly(3-hydroxybutyrate-co-3-hydroxyvalerate) (PHBV) resulted in a biomimetic composite, which is able to form CaP crystals within the whole scaffold, with PHBV content strongly affecting its chemical composition and surface characteristics, in turn affecting the cytocompatibility [163]. On the other hand, gelatin modified with CaP nanoparticles and PCL was used to prepare a 3D bi-layer scaffold by collecting electrospun PCL and gelatin/CaP fibers separately in the same collector to enhance mineralization, thus improving the ability of the scaffold to bond to the bone tissue [164]. The use of PCL and CaP nanoparticles resulted in a synergistic effect: On the one hand, the PCL improved the mechanical properties of the scaffold, whereas the addition of CaP nanoparticles enhanced its bioactivity, which is attested by higher ALP activity with human osteoblast cells.

**Figure 3:**
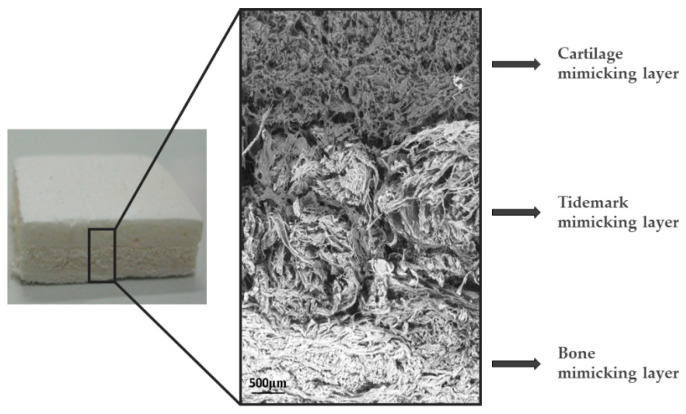
Three-dimensional hybrid scaffolds by bio-inspired mineralization processes for regeneration of osteocartilaginous tissues (**left**) and SEM micrograph showing multi-layered structure (**right**).

Previous studies highlighted the possibility of inducing the heterogeneous nucleation of bioactive apatitic crystals on natural polymers by reproducing the cascade of phenomena occurring in vivo during new bone formation, with the purpose of obtaining scaffolds which are able to trigger bone regeneration processes only by virtue of their compositional, morphological, and ultrastructural properties [165]. These bio-inspired mineralization processes involved the use of bio-polymers, particularly type-I collagen fibrils, which are dispersed in an aqueous solution of ions generally implicated in bone formation processes, the amount of which can determine the extent of the mineral phase in the bio-composite. An interesting aspect is that in these hybrid constructs the mineral phase is not simply embedded, but heterogeneously nucleated on a bio-organic matrix, thus more closely mimicking the ultrastructural features of bone tissue. In this context, fibrous hybrid materials could also be obtained as a multilayered device reproducing the different mineral content present in multifunctional tissues, such as osteochondral or periodontal regions (Figure 3) [165,166,167,168]. This approach was intended to respond to a relevant clinical need related to the repair of osteochondral defects [169,170] using a 3D biomimetic scaffold that shows at the same time bioactive composition, porous fibrous structure, and good malleability, which is able to mimic the different osteochondral regions, namely bone, tidemark, and articular cartilage.

As natural polymers are easily subjected to fast enzymatic dissolution, the resorptive properties of hybrid scaffolds can be modulated using specific cross-linking additives, such as 1,4-butanediol diglycidyl ether (BDDGE), genipin or ribose, which are suitable for modulating the strength of interfibrillar bonds and in turn, the bioresorbability, hydrophilic properties, and mechanical performance of the bio-organic template [171,172,173]. In the last decade, the nucleation of apatite crystals in the presence of agar-gelatin hybrid hydrogel have been examined to understand the cooperative effects of the organic templates on the formation of the inorganic apatite phase, with favorable effects on the proliferation and differentiation of osteoblast-like MC3T3-E1 cells [174,175]. This system is suitable for the study of bio-inspired mineralization processes since (a) gelatin could be incorporated into the hydrogel and remain stable during the experiment, (b) the local concentration for crystallization is readily achievable, and (c) the deposits are easily harvested from the medium without agar contamination.

Bio-inspired hybrid materials possess excellent abilities in bone defect regeneration and also offer the possibility of developing new promising alternatives for the regeneration of osteochondral defects [176]. The regenerative ability boosted by high mimicry of osteochondral regions was attested by an in vivo study with collagen/apatite hybrids on sheep, showing that the various layers of the scaffold induced specific cell differentiation into osteoblasts (in the bony region) and chondrocytes (in the cartilaginous region) with the formation of osteochondral tissue with ordered histoarchitecture [177]. Hybrid collagen/apatite osteochondral scaffolds were also applied in various clinical trials. In most cases, the original functional structure of the cartilage was recovered within 2 years, as attested by numerous bio-markers that report the successful remodeling of the original fibrocartilage tissue into hyaline cartilage [178,179,180,181]. A noticeable aspect of the biomimetic approach, confirmed also by clinical studies, is the attainment of devices that are able to modulate the cell behavior only, thanks to physicochemical and structural features, without using any added growth factors. However, these hybrid, fibrous structures generally lack mechanical properties, enabling their application in load-bearing regions in order that technological advances in the incoming decades are highly desired and expected, in the perspective of reducing the recourse to invasive metallic joint reconstruction prosthesis, which is currently the only available solution for patients affected by osteochondral diseases.

### 5.3. Bioactive Glass Scaffolds

The development of silica glassy phases is among the first approaches considered to generate bioactive ceramics for bone regeneration. Bioactive glass structure is primarily composed of an interconnected open network of SiO_4_ tetrahedra that does not possess a uniform arrangement. This peculiar state favors the insertion of cations of various nature, referred to as network modifiers. The disruption of the O-Si-O network caused by the insertion of cations, such as Na^+^, K^+^, and Ca^2+^ and subsequent formation of non-bridging oxygen moieties result in high surface reactivity of these materials in an aqueous environment. Insertion of network modifiers, such as CaO and Na_2_O in a network comprising SiO_2_ and P_2_O_5_ is one of the most studied formulations, which gives rise to the formation of bioactive glasses with many applications in bone tissue engineering, as the crystallization of apatite-like phases is obtained on the surface of the glassy phase [182]. Magic-angle spinning nuclear magnetic resonance (MAS-NMR) spectroscopy has been applied for the study of the surface reaction of mesoporous bioactive glass (MBG) immersed in simulated body fluid (SBF) coupled with XRD analysis, proving the formation of amorphous calcium phosphate and subsequent crystallization into carbonated apatite within the first 4 to 24 h of SBF exposure, respectively. After 1 week, nanocrystalline carbonated apatite constitutes the main fraction (≈ 60%) of the total amount of phosphorus-bearing phases [183]. The ability of bioactive glasses to bond to bone tissue is a result of their chemical reactivity in physiological media and involves multiple steps:

1. Leaching through the exchange of protons from the physiological medium with labile network-modifying ions, such as Na^+^, K^+^, Ca^2+^, Mg^2+^, etc.:

Si − O − Na^+^ + H^+^ + OH^−^ → Si − OH^+^ + Na^+^(solution) + OH^−^

The cation-exchange process increases the concentration of hydroxyl ions at the bioactive glass–solution interface, thereby raising the pH.

2. The previous pH rise facilitates dissolution of the network and formation of additional silanol groups according to the reaction:

Si − O − Si + H_2_O → 2 [Si − OH^+^]

as well as the loss of soluble silica as Si(OH)_4_ passes into the solution.

3. Polymerization of the SiO_2_^−^ rich layer through condensation of neighboring Si–OH groups, which produces a layer rich in amorphous silica.
4. Migration of Ca^2+^ ions to the surface of the silica-rich layer to form an amorphous film rich in CaO–P_2_O_5_, followed by thickening of the film by incorporation of soluble Ca^2+^ and PO_4_^3−^ ions from the solution.
5. Crystallization of the amorphous CaO–P_2_O_5_ film by incorporation of OH^−^ and CO_3_^2−^ from the solution, to form a carbonated apatite [184,185].

The controlled release of ions from bio-glasses in this context provides chemical signals and soluble Si and Ca ions are believed to be critical for controlling osteogenesis and/or activating genes responsible for osteogenesis in biological environment [186]. Studies have shown that MBGs do not inhibit osteoclastogenesis and allow for macrophage proliferation without inducing polarization toward M1 pro-inflammatory phenotype, indicating that MBGs would allow for the innate immune response required for the healing process without further inflammatory complications [187,188].

A major drawback in the development of bio-glass scaffolds is linked to the difficulty in assembling these materials into 3D scaffolds with appropriate mechanical properties. Indeed, upon heat treatment suitable for consolidation processes, bioactive glasses tend to undergo transformation into crystalline phases. Therefore, various attempts have been made to design fabrication strategies focused on the optimization of the glassy phase composition (for instance, by increasing the calcium/alkali ratio and partially replacing sodium with potassium) [189] to increase the crystallization temperature. In a different approach, glassy phases were used in the mixture with polymers to obtain reinforced composite materials [190,191,192]. As previously stated, the use of polymers in the design of biocompatible scaffolds has intrinsic problems related to degradation products, which are formed during bone resorption processes. However, the use of Bioglass^®^ as an additive to PLGA foams has been reported to provide pH buffering effects at the polymer surface, which is assessed by long-term incubation tests (i.e., 30 days) [193,194], thus promising to reduce the drawbacks related to the use of polymers in bone scaffolds.

### 5.4. Self-Hardening Apatitic Scaffolds

When designing a 3D scaffold for bone substitution, it is necessary to consider the limitations imposed by the clinical procedure. Although most of the biomaterials designed to date have outstanding mechanical and biological properties after in vitro and in vivo trials, effective clinical applications are often limited by the difficulty in adapting the scaffold to complex shape defects or in accessing specific anatomical districts, such as for instance, the spine or the femur head or even by fixation problems [195]. Since in the area of regenerative medicine and dentistry CaPs have been already largely investigated due to their biocompatibility and osteoconductive behavior, new self-hardening materials, such as calcium phosphate cements (CPC) have been developed since decades, showing the ability to perfectly adapt to hard tissue defects (tooth, bone, etc.) [196].

Generally, CPC formulation involves the use of combination of calcium orthophosphates, which upon mixing with aqueous solutions forms a paste, which is able to harden after the implantation process at body temperature in the targeted area. Setting processes are primarily the result of dissolution processes involving the reagents and the precipitation of the final product. Classical CPC that employs α-tricalcium phosphate as the active reagent yields the formation of hydroxyapatite as a consequence of the hydrolysis reaction:

$$3\alpha -{\mathrm{Ca}}_{3}{\left({\mathrm{PO}}_{4}\right)}_{2}+{\;H}_{2}O\;\to {\mathrm{Ca}}_{9}\left({\mathrm{HPO}}_{4}\right){\left({\mathrm{PO}}_{4}\right)}_{5}\left(\mathrm{OH}\right)$$

Alternatively, other types of CPC rely on acid-base reactions that entail the use of alkaline tetra calcium phosphate (TTCP) mixed with acidic di-calcium phosphate anhydrous (DCPA) following the reaction:

$${\mathrm{Ca}}_{4}{\left({\mathrm{PO}}_{4}\right)}_{2}O\;+{\;\mathrm{CaHPO}}_{4}\to {\mathrm{Ca}}_{5}{\left({\mathrm{PO}}_{4}\right)}_{3}\left(\mathrm{OH}\right)\;$$

**Figure 4:**
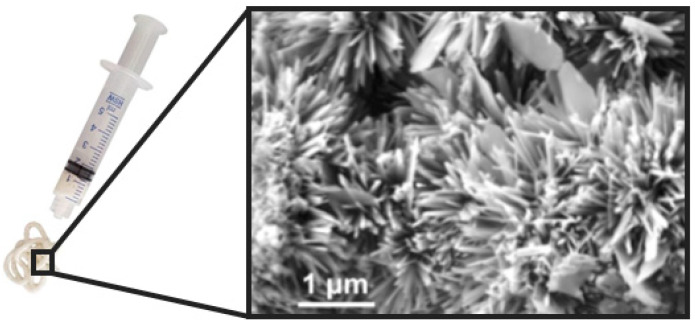
Biomimetic injectable bone cements (**left**) and SEM micrograph showing the typical needle and plate-like morphology (**right**).

Other cement formulations provide different reaction products upon setting, such as brushite (CaHPO_4_·2H_2_O). As the hardening process occurs in a biological environment, the resulting apatite has strong similarities to biological apatite in terms of low crystallinity, high specific surface, bioactivity, and bioresorbability [45]. The peculiar morphology and hardening process rely on the physical interlocking of elongated HA crystals that form upon the hydrolysis process, thus resulting into micro/nanoporous architectures (Figure 4). What designates CPCs as versatile products are the plentiful variables that can be altered to deliver final products with specific rheological and mechanical properties. Main parameters affecting the porosity are, for example, the liquid/solid ratio and the particle size of the reagents used, that influence substantially the dissolution/precipitation processes [195,197]. The insertion of additives, such as biocompatible mannitol, for example, assisted the fabrication of bi-porous apatite without any inhibitory effects on the transformation into apatitic phases [198]. Although promising for bone regeneration purposes, it is widely accepted that CPCs suffer to satisfy clinical requirements, such as difficulty in obtaining good injectability and cohesion, as well as a general lack of mechanical strength, toughness, and brittleness, limiting their application in load-bearing defect sites [10,196]. The study of the CPCs scaffold evolution in biological environment highlights additional problems, such as the disintegration of the CPC paste upon early contact with biological fluids, such as blood due to weak cohesion and low porosity, which hinders homogeneous colonization of bone resorbing cells, compromising bioresorbability of the resulting scaffold, and limiting their extensive use in orthopaedics [196].

Studies aimed at the improvement of the mechanical properties and control over the biodegradability and scaffolding technique, such as macropore generation, involved the introduction of a variety of degradable polymers. Incorporation of sodium alginate caused a general decrease in setting behavior with different power/liquid ratios and increase in compressive and tensile strength of the hardened biomaterial [199]. The employment of other types of biopolymers, such as hyaluronic acid [200,201] and chitosan [202] revealed promising effects on cement injectability, making CPCs a viable example of bone filler for tissue engineering.

As the formation of HA and subsequent hardening process takes place at room temperature, it is possible to incorporate in the paste formulation drugs or other bioactive molecules, with the potential to act as a multifunctional drug carrier and delivery systems. In this field of study, antibiotics (such as gentamicin sulfate [203,204] and tetracycline hydrochloride [205,206]) have been studied in the prevention of postoperative bacterial infections. In addition, local control of drug release could be very useful in the treatment of different skeletal diseases, such as bone tumors, osteoporosis or osteomyelitis, since in CPCs drugs can be homogeneously combined in at least one of the cement phases, making the controlled release process in body fluid prolonged in time [207,208,209].

### 5.5. Mechanically Bearing, Biomorphic 3D Scaffolds

As previously stated, bone mechano-transduction process is a biologic phenomenon, which is able to effectively translate external mechanical stimuli exerted on the skeletal system into bio-electric signals, that can instruct bone cells to activate and sustain the continuous bone remodeling and self-repairing upon damage. Therefore, its activation is very important when it comes to regenerating the load-bearing bone parts. However, the reproduction of the complex biomechanical ability of bone tissue is quite a challenge. To date, it prevented the achievement of scaffolds which are effective in healing load-bearing bone defects [210].

A relevant example is the case of segmental bone defects (injuries in which a section of bone is completely shattered and/or absent), which the body cannot heal on its own. The common surgical procedures refer to the use of metallic components or bone bank pieces, which however cannot assure the regeneration of long bones with all its biomechanic functions and often results in adverse complications, such as pseudo-arthrosis, malunions, and loss of function [211,212,213].

A major limiting factor arises from insufficient vascularization, particularly when large bone defects have to be treated, which could ultimately lead to inefficient nutrient supply in the inner regions of the scaffold causing bone necrosis or insufficient cell penetration [147,214,215]. This drawback is the consequence of scaffolds with inappropriate composition favoring cell conduction, and/or insufficient pore structure and interconnection. In this context, novel approaches in scaffold development increasingly look at nature and involve the use of biostructures as sacrificial templates to fabricate advanced materials with morphological features replicating those of living organisms [216,217]. In this context, the great abundance of biologic structures with outstanding mechanical performance is a unique source of inspiration for material scientists for the production of implantable devices by structural replication of these natural sources.

Aragonite CaCO_3_-based crystals at the basis of the skeleton of common scleractinian, reef-building, colonial coral Porites (total porosity below 60 vol%, pore size range within 140–160 µm with all the pores interconnected), and Goniopora (total porosity above 70 vol%, large pore size ranging from 200 to 1000 µm) have been studied as a suitable template for the formation of biomorphic scaffolds with controlled porosity through the so-called “replamineform process” (meaning “replicated life forms”) [218]. The general synthesis of coral-derived biomorphic apatites (BA) involves the thermal treatment and decomposition of CaCO_3_ into CaO, followed by chemical exchange with (NH_4_)_2_HPO_4_ that leads to the conversion into HA, as the most thermodynamically stable CaP phase. Although this method allowed for the production of scaffolds with controlled micro-architecture, coral-derived HA implants developed to date were associated with brittleness, thus preventing effective surgical procedures. Indeed, it was shown that the unique mechanical performance of bone tissue is strongly related to its multi-scale hierarchic structure, which is lacking in most of the conventional ceramic devices, including coral-derived ones, as corals have a structure quite different from that of bone.

**Figure 5:**
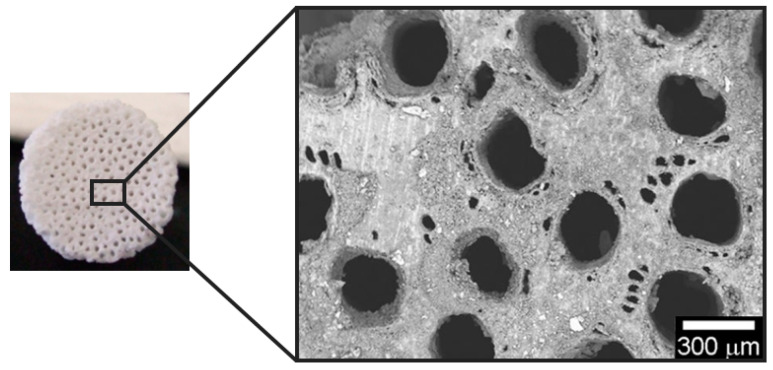
Hierarchically organized biomorphic scaffolds for regeneration of load-bearing bones (**left**) and SEM micrograph showing the macroscopic longitudinal channels mimicking the osteon (**right**).

In this context, suitable alternatives were found for the vegetable kingdom, particularly wood structures. The macroscopic architecture of wood materials is generally composed of an alternation of fiber bundles tightly aligned and channel-like porous areas, with 3D structures strongly resembling the one observed in compact bone. The conversion of wood material into biomorphic scaffolds can be achieved through pyrolysis of the wood organic structure, which is principally composed of cellulose, hemicellulose, and lignin, followed by the infiltration of one of the precursors that give rise to the consolidated ceramic material [219]. Drawbacks related to infiltration methods are mainly related to the need of a final sintering for the scaffold consolidation, thus preventing the achievement of bioactive composition and nanoporosity, which is relevant for fluid exchange in vivo. In the last decades, studies have been aimed at the improvement of the chemical processes at the base of the synthesis of novel scaffolds, which are composed of biomimetic HA. This employs rattan wood as a template to guide a complex chemical process based on heterogeneous chemical reactions, which occurs in the 3D state rather than by infiltration of inorganic precursors. This approach permitted the translation of the hierarchic rattan wood structure, closely mimicking the osteon system of compact bone, with its outstanding mechanical properties, to these 3D scaffolds with a composition consisting of ion-doped nanocrystalline apatite (Figure 5). In bioreactor studies, these scaffolds enhanced osteogenic and angiogenic differentiation, which is attested by the outstanding upregulation of genes involved in both early (BMP2, Runx2, and ALP) and late (OPN and Col15a1) stages of osteogenic commitment, when compared with the sintered apatite [10], as well as enhanced in vivo osteoinductive ability upon testing in rabbit, particularly well-developed bone tissue, which is formed in ectopic site 12 weeks after subcutaneous implantation [213]. Moreover, the structural hierarchy determined mechanical performances that are unusual for pure ceramic materials in terms of damage tolerance, strength, and elastic stiffness, which are superior with respect to porous sintered HA, but also to other biomorphic apatites previously obtained with different types of woods and processes [220,221,222,223].

Biomorphic apatite scaffolds derived from rattan showed an ability to completely regenerate metatarsal long bone defects in sheep, showing the complete bio-resorption of the scaffold which allowed the well-organized bone with mechanical properties to reproduce the original bone tissue [224]. Regeneration of load-bearing segmental bones is among the most relevant challenges considered in orthopedics. Therefore, this result confirms that heterogeneous chemical reactions are a promising approach to transform natural structures into 3D nanostructured apatitic scaffolds, recapitulating the major properties relevant for the inducement of bone regeneration, in terms of composition, porosity, structure, and mechanics.

## 6. Conclusions and Future Perspectives

The development of materials and devices for tissue regeneration is an interdisciplinary field that still presents several challenges. As novel effective solutions are still required, the application of the biomimetic concept is gaining ground as relevant for inspiring the development of scaffolds, as well as more effective in reducing adverse reactions, drawbacks, and improving the bio-functionality and regenerative ability. This is particularly relevant in the case of the bone tissue, which exhibits great compositional and structural complexity, at the basis of its outstanding mechanical performance and biological activity. In this context, the need of sintering processes for ceramic scaffold consolidation is a major aspect that prevents to date the development of effective clinical solutions. Nowadays, various new approaches based on low temperature processes shed new light on the possibility of obtaining 3D scaffolds with synergistic biomimetic features, as related to composition, structure, and mechanics. This is promising for the resolution of various, still unmet, clinical needs of great societal relevance, particularly bone regeneration, considering the relevance of healthy skeletal tissues for human life and well-being. On the other hand, bone regeneration is a problem that strongly depends on the specific anatomical site (e.g., whether in the cranium, limbs or spine), characterized by different functional and mechanical demands. Therefore, every specific case should be investigated by a clinical perspective to determine the best technological approach for the achievement of the most appropriate solution for that specific case.

## Figures and Tables

**Table 1:** Main ions doping bone mineral and their biological effects.

Ion Substitution Site	Doping Ion	Main Effects
Ca^2+^	Mg^2+^	Magnesium ion is quantitatively the most important, typically amounting to around 6 mol%. In biological environment, magnesium boosts skeletal metabolism and bone growth, and its deficiency adversely affects all stages of skeletal metabolism, causing a decrease in osteoblastic and osteoclastic activities, osteopenia, and bone fragility [88,89].
	Sr^2+^	Strontium ion increases bone formation, the number of bone-forming sites, and bone mineral density, and reduces bone resorption, leading to a gain in bone mass and improved bone mechanical properties in animals and humans [90]. Strontium increases osteoblast activity and decreases osteoclast resorption, making it suitable for the treatment of osteoporosis [91].
	Zn^2+^	Zinc ion stimulates osteoblastic activity in vitro and inhibits bone resorption in vivo [45]. In addition, doping induces the segregation of bioactive ions on the material surface which makes them available for exchange with physiological fluids, preventing bacterial antibiotic resistance in hospitals during the postoperative period [86].
PO_4_^3−^	SiO_4_^4−^	Silicates are among the trace elements in HA involved in biological processes. SiO_4_^4−^ substitution of phosphate ions site charge difference causes the formation of a Ca^2+^ partial vacancy for the equilibration of charge neutrality. Silicates enhance osteoblast cell proliferation compared with the pure HA phase and its depletion is often related to the deterioration in the proliferation and function of osteoblast due to osteoporosis and osteopenia [92,93,94].
	CO_3_^2−^	The substitution of phosphate groups with carbonate ions is called B-type carbonation. B-type carbonation is present in young bone, which is subjected to remodeling processes, resulting in higher solubility [89,95].
OH^−^	Cl^−^	Chlorine ions in HA structure provide an acidic environment on the surface of bone that stimulates osteoclasts in the bone resorption process. Accordingly, this incorporation may be essential in the expansion of low pH to solubilize the alkaline salts of bone minerals and to digest the organic matrix by acid hydrolases, which are secreted by osteoclasts [96,97].
	F^−^	Fluorine ions substitution provides higher chemical and thermal stability. Moreover, the fluorine ion itself is known to suppress dental caries and stimulate the proliferation and differentiation of bone cells [97,98,99,100].
	CO_3_^2−^	The carbonation of the hydroxyl site is called A-type substitution. Studies have found that biological apatites, such as dentin, phytolith, and dental calculus have an A–B mixed type carbonation (B > A), and kidney stones may be both A–B mixed and B-type [101]. In bone apatites, A-type carbonation concentration progressively increases with age and maturation as the resulting apatite is less soluble and less subjected to remodeling processes [89,95].

## Data Availability

Not applicable.
